# Epstein-Barr-Virus-Positive B-Cell Lymphoma of Recipient Origin Despite of the Elimination of Clonally EBV-Infected T Cells by Allogeneic Stem Cell Transplantation in a Patient with Chronic Active EBV Infection

**DOI:** 10.1155/2012/164824

**Published:** 2012-06-13

**Authors:** Masayuki Nagasawa

**Affiliations:** Department of Pediatrics and Developmental Biology, Graduate School of Medical and Dental sciences, Tokyo Medical and Dental University, 5-45, Yushima 1-chome, Bunkyo-ku, Tokyo 113-8519, Japan

## Abstract

A 20-year-old patient with chronic active EBV infection (CAEBV) received peripheral blood stem cell transplantation (PBSCT) from HLA-one-locus-mismatched mother. Although EB-virus-infected T cells were eliminated after PBSCT, she developed EB-virus-positive B-cell lymphoma of recipient origin in the brain. By reducing the immunosuppressive therapy, the initial lesion disappeared. However, another lesion in the opposite lateral brain appeared later and was resistant to further reduction of immunosuppressive therapy. EBV-DNA was persistently negative after PBSCT in the peripheral blood. This case is suggestive in management of EBV reactivation after SCT and understanding alloimmune response to EBV.

## 1. Introduction

Epstein-Barr virus (EBV) is a ubiquitously spread herpes family virus, which infects more than 90% of adults [1]. Primary EBV infection often is asymptomatic and sometimes presents as acute febrile mononucleosis. EBV usually infects B cells and latently infected B cells are regulated by potent T cells [2]. In an immunodeficient state, these B cells occasionally induce lymphoproliferative disease, which is sometimes life threatening [3–5].

Chronic active EBV infection (CAEBV) is another EBV-related lymphoproliferative disease in which EBV infects T or NK cells [6, 7]. Although the pathogenesis is not well understood, the prognosis is poor in the long run and allogeneic stem cell transplantation (allo-HSCT) is recommended to eradicate infected T or NK cells [8]. With an increased transplantation-related mortality by conventional myeloablative conditioning regimen (MAC), reduced intensity conditioning regimen (RIC) has been applied, resulting in an improved 3-year overall survival of 95.0 ± 4.9% in RIC compared to 54.5 ± 15.0% in MAC [9]. Recently, the author has reported that level of serum granulysin is useful for discriminating NK cell type CAEBV from T cell type [10].

The author has experienced a patient with CAEBV who developed EBV-positive B-cell lymphoma of recipient origin after allogeneic stem cell transplantation. The clinical course of this patient is suggestive to understand the significance of allo-immune response against EBV-associated lymphoproliferative diseases (EBV-LPD) in the setting of allo-HSCT.

## 2. Case Report

**Figure 1:**
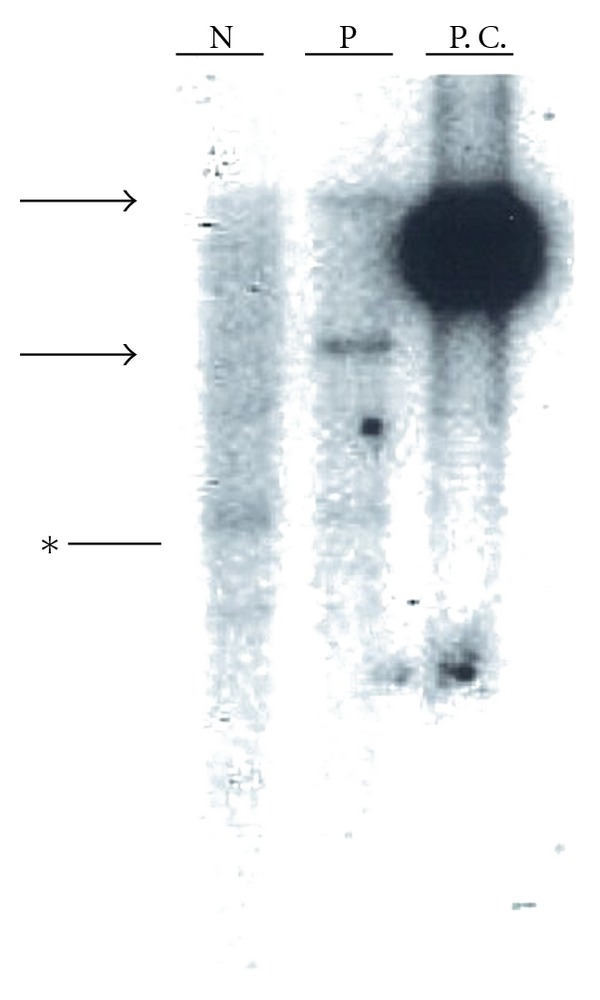
Infection status of EBV was monoclonal. DNA from the patient's peripheral blood mononuclear cells was digested by EcoRI, and southern blot hybridization was performed with EBV BamHI W probe. An arrow points at the monoclonal bands. Asterisk shows the nonspecific band. N: normal control; P: patient; P.C.: positive control (Raji cells).

The patient presented with mosquito hypersensitivity at the age of five. Thereafter, she had recurrent cervical lymphadenopathy with fever, which resolved spontaneously in a couple of weeks. When she was 14 years old, systemic lymphadenopathy with fever developed. Biopsy of cervical lymph node revealed the diagnosis of Hodgkin lymphoma. She received chemotherapy of COPP/ABVD and modified EPOCH for two years. However, febrile lymphadenopathy resumed after chemotherapy. Then, she was diagnosed as chronic active EBV infection from the persistent presence of increased EBV-DNA (10^4^–10^5^ copies/mL) in the peripheral blood and high titer of anti-EB virus antibody. Analysis of lymphocyte subsets revealed that EBV infected both *αβ*T and *γδ*T cells and they were expanded clonally (Figure 1).

She was referred to our hospital at the age of 20. She received peripheral blood stem cell transplantation (PBSCT) from HLA-one-locus-mismatched mother at the age of 21. Conditioning regimen included fludarabine (25 mg/m^2^  × 5), melphalan (70 mg/m^2^  × 2), and antithymoglobulin (Lymphoglobuline; 10 mg/kg × 2). GVHD prophylaxis was tacrolimus with short-term MTX. Engraftment was on the 9th day, and grade III GVHD (skin: stage 3, liver: stage 2) occurred on the 14th day, for which prednisolone and mycophenolate mofetil (MMF) were started. GVHD was intractable and grade II GVHD (skin) persisted. On the 198th day, she was discharged for follow-up of chronic GVHD on medication of tacrolimus (0.1 mg/kg/day), prednisolone (0.5 mg/kg/day), and MMF (50 mg/kg/day). EBV-DNA in the peripheral blood was turned negative on the 10th day. On the 40th day, it became transiently positive but was consistently negative after the 52nd day. 

**Figure 2:**
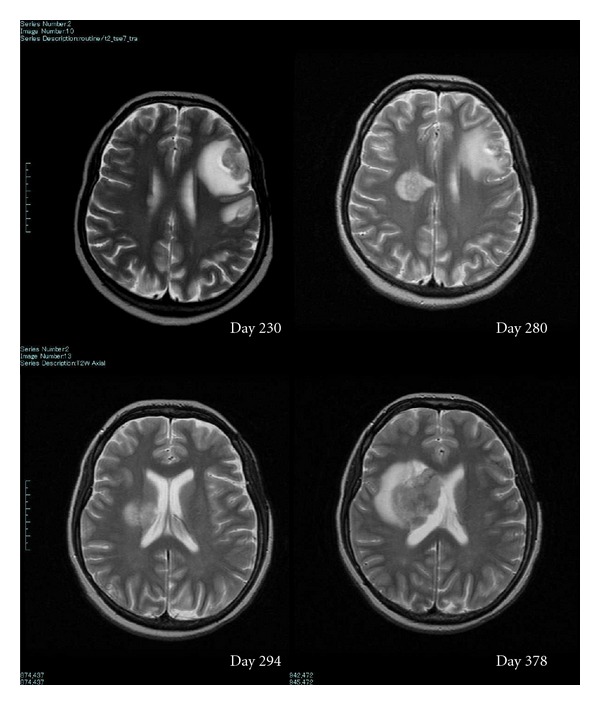
Brain MRI of the patient. Initial frontal tumor that was surrounded by T2 high edematous area disappeared completely on the 294th day, but right paraventricular tumor grew progressively.

On the 227th day, she was transferred to our hospital because of convulsion. MRI revealed a mass with diameter of 20 mm in the left frontal lobe (Figure 2). EBV, CMV, VZV, and HHV-1, 2, 6, 7, 8 were all negative in both of the peripheral blood and spinal fluid by PCR method. Considering the EBV-associated lymphoma or toxoplasmosis, immunosuppressive drugs were reduced and antitoxoplasma drug (pyrimethamine + sulfadiazine) was started. Stage 2 skin GVHD developed, and left temporal mass was gradually regressed. However, MRI on the 280th day disclosed another mass in the right paraventricular area (Figure 1). EBV-DNA was detected in the spinal fluid on the 373rd day, although still negative in the peripheral blood. The mass was progressively enlarged in spite of stopping tacrolimus and MMF. She and her family were reluctant to further intensive therapy including radiotherapy. She died from brain stem herniation on the 417th day after PBSCT. 

**Figure 3:**
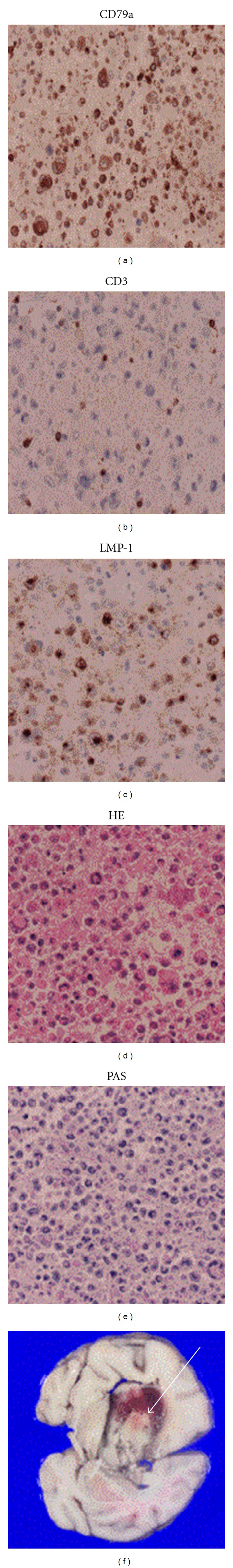
Histology of the brain tumor. Infiltrating cells in the brain tumor were CD79a+, CD3−, and LMP-1+. Arrow indicates the right paraventricular tumor.

**Figure 4:**
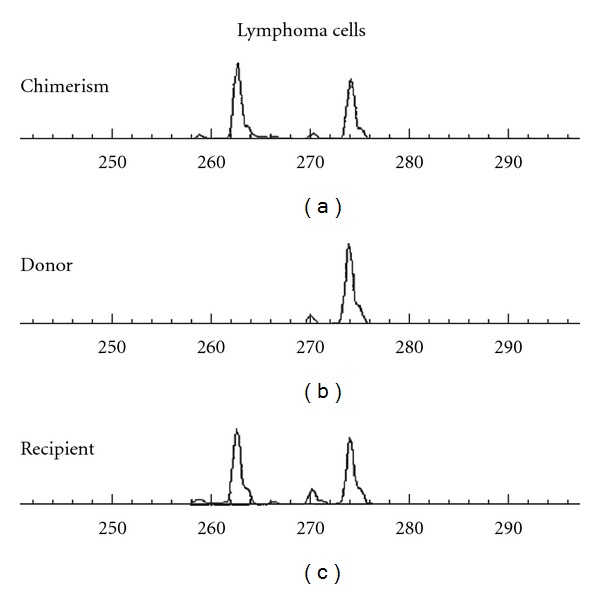
STR (short tandem repeat) analysis of the lymphoma cells.

Autopsy revealed EBV-positive diffuse large cell lymphoma positive for CD20 and CD79a (Figure 3). Analysis of IgH and EBV terminal repeat presented monoclonal proliferation and STR (short tandem repeat) analysis showed the recipient origin (Figure 4). In the left hemisphere, ghost cells were aggregated where the first mass was detected. Ghost cells were positive for CD20 and LMP-1 (latent-membrane-protein-1) and considered to be apoptotic lymphoma cells. There was no evidence of toxoplasmosis histologically.

## 3. Discussion 

It has been reported that EBV reactivation occurs in 0.6 to 26% after allo-HSCT, with this being higher in the context of T-cell depletion or prolonged immunosuppressive treatment for intractable GVHD. EBV-LPD typically occurs within the first 6 months after allo-HSCT, and mortality rates can range between 50 and 80% [11]. In most of the cases, EBV-LPD arises from donor lymphocytes in allo-HSCT contrary to our case. To avoid the development of EBV-LPD, weekly screening of EBV-DNA for at least 3 months and early preemptive intervention with rituximab is recommended when EBV-DNA exceeds 10^3^ copies/10^5^ cells in the peripheral blood for high-risk patients [12].

It is quite interesting that EBV-DNA was persistently negative in the peripheral blood after PBSCT in this patient, and it finally turned positive only in the spinal fluid at the later stage. This observation suggests that serial monitoring of EBV-DNA in the peripheral blood could not be sufficient enough for the detection and evaluation of EBV-LPD in the central nervous system (CNS). Isolated CNS EBV-LPD is extremely rare, and only five cases have been reported in the medical literature so far. In one case, EBV-DNA was also negative in the spinal fluid by PCR method as our case [13]. 

In this patient, EBV-infected *αβ*T and *γδ*T cells were eliminated after allo-HSCT, which was confirmed by the disappearance of EBV-DNA from the peripheral blood. However, EBV-positive B cell lymphoma was induced under immunosuppressive treatment for chronic GVHD. 

In this case, allogeneic immune response was considered enough to eliminate EBV-infected monoclonal T cells but not EBV-infected monoclonal B cells. One possibility is that the central nervous system was completely separated from allogeneic immune response. However, with the fact that initial brain tumor regressed after reducing immune suppressive drugs, this scenario seems not enough to explain the disease progress. Although not investigated precisely, the initial lesion in the left hemisphere could be biologically different from that in the right brain because the former regressed by reducing the immunosuppressive drugs. Recently, it has been reported that loss of alloantigen is associated with the resistance to immune surveillance system in malignant hematological disease [14]. Another possibility is that the latter lymphoma cells were more aggressively transformed to be resistant to apoptotic stimuli.

Although we could not investigate the underlining molecular mechanism in the different resistance for each lymphoproliferative disease to alloimmunity in detail, this case seems suggestive to consider the efficacy and limit of cellular immune response to EBV-related lymphoproliferative disease.
